# Viewpoint diversity in public health

**DOI:** 10.3389/fpubh.2023.1263767

**Published:** 2023-08-31

**Authors:** Tyler J. VanderWeele

**Affiliations:** Departments of Epidemiology and Biostatistics, Harvard T. H. Chan School of Public Health, Boston, MA, United States

**Keywords:** academic freedom, population health, universities, viewpoint diversity, values

## Abstract

Schools of public health are often situated within universities but not infrequently also function as public health advocacy organizations. Viewpoint diversity on many issues is often limited within schools of public health and does not reflect the diversity one finds in society more generally. It is argued that welcoming, and even seeking out, viewpoint diversity within public health would contribute to understanding and knowledge, to the training public health leaders and academics who can serve the whole of society, and to working together across ideological lines to better contribute to population health.

## Introduction

Schools of public health and medical schools often constitute academic units within a university, but sometimes also function as health advocacy organizations. Faculty are of course generally free to advocate for various positions, but sometimes such advocacy takes place at the level of the School as well, as when official School communications clearly take specific positions on controversial issues (1–3). Tensions can sometimes arise between these academic and advocacy functions; decisions then need to be made as to how to structure the institution, and how to treat its members. It is not clear, for example, the extent to which those who hold minority perspectives on certain issues are allowed to express them within public health and still be treated civilly. Over the past months, the fact that I hold a number of unpopular minority viewpoints on controversial moral issues caused turmoil at the Harvard T. H. Chan School of Public Health (4, 5). Others may hold positions that constitute minority viewpoints within academic public health because of different political commitments, religious commitments, or simply through independent thought. This raises important questions concerning who is welcome to participate in academic public health, and in what manner. In this commentary, I would like to briefly offer thoughts on these questions, to argue for the value of intellectual diversity in academic public health, and to raise a number of the issues that I have discussed in greater and more personal detail elsewhere (4). Specifically, I will argue first that welcoming viewpoint diversity would contribute to the pursuit of knowledge; second, that there should be greater clarity within schools of public health as to which ideas, principles and beliefs, if any, are to be excluded because they incompatible with the mission of a school; and third, that welcoming viewpoint diversity will facilitate the training of public health leaders and academics who are able to serve the whole of society.

## Universities, knowledge, and academic public health

Modern research universities are characteristically oriented towards the generation, preservation, and dissemination of knowledge. Achieving this purpose requires the capacity for free inquiry, robust debate, and the weighing of evidence and arguments. This in turn is facilitated by the university being a forum in which a broad range of ideas and beliefs, even those which may be strongly at odds with one another, are welcome (6–8). The members of an academic community have a responsibility to put forward reasoned arguments, but different people come with different starting points, values, and presuppositions. The process of rational discourse is in part meant to uncover those presuppositions, and to evaluate the extent to which logic and evidence supports a given conclusion. The value of this diversity and free exchange of ideas is that, by engagement with other perspectives, one understands others’ beliefs and thinking better; one can often understand and refine one’s own views; others can learn from debates; and the community is strengthened in its pursuit of knowledge and in its confidence that knowledge has in fact been attained (6–10). We can conduct our discussions and arguments respectfully, with the recognition that others will often disagree with us, and may do so passionately. Through civil discourse, our understanding of our own and others’ ideas can be sharpened and we can sometimes find common ground. Free inquiry, civil discourse, intellectual diversity, and rational debate are all means. Knowledge and understanding are the ends.

Within academic public health, the promotion of population health will also be considered an important end. This can sometimes also alter the value placed on civil discourse, intellectual diversity, and rational debate. If these academic values are seen as in tension with professional and public health objectives in certain cases, these academic values might be rejected. Some may view the notion of rational discourse as one of many attempts to grab power. Others may disparage the notion of respectful discourse as itself potentially a tool of oppression. Some may reject the notion of finding common ground, as it may seem to compromise the strength of one’s position. From the perspective of public health advocacy with a particular agenda, these alternative viewpoints are themselves perhaps understandable. An approach which rejects reasoned engagement, civil discourse, and finding common ground may sometimes be the most expedient way to one’s end. It may also sometimes be administratively easier not to defend such academic principles and values when challenged. However, their rejection detracts from a university’s purpose to generate, preserve, and disseminate knowledge; it instead alters that purpose for different political ends. It is also less clear that rejecting these values of civil discourse offers much hope for the future of a pluralistic democracy. How are we to navigate disagreements within society if we have not first engaged in the work of understanding alternate perspectives?

Freedom of expression can certainly be abused within a university and within academic public health and there are risks to granting such freedoms (9), but by treating one another civilly and respectfully we can try to prevent those abuses. Moreover, without taking the risk of guaranteeing those freedoms, there is potentially a severe loss with regard to our own capacity to pursue knowledge. The loss is arguably well-characterized by John Stuart Mill, in his work *On Liberty* (10): “He who knows only his own side of the case knows little of that. His reasons may be good, and no one may have been able to refute them. But if he is equally unable to refute the reasons on the opposite side, if he does not so much as know what they are, he has no ground for preferring either opinion.” Without engaging with differing viewpoints it can be more difficult to see gaps in evidence, or to see countervailing evidence, or alternative interpretations of results, or when a position in fact concerns values rather than science (11).

## Viewpoint diversity in academic public health

The question of which viewpoints to admit within academic schools of public health and medicine depends in part on whether these institutions are being principally conceived of as parts of the university and, hence, as institutions that promote reasoned debate across individuals and groups with diverse beliefs and goals. Viewpoint diversity within universities is often desirable; viewpoint diversity within health advocacy is more complex. It would, however, be good to have greater clarity as to which beliefs, moral principles, and positions are to be considered admissible, and which are thought beyond the pale.

Are the positions of all elected members of congress to be admissible within academic public health discussion in the United States? Or only the most centrist 80% or 90%? Or only those sufficiently far left? Should it be permissible to silence or exclude minority positions that are held by 10% or 30% or nearly 50% of the American population? Clarity on such issues would help address the question of the extent to which a particular institution considers it acceptable to express viewpoints on moral controversies that constitute minority positions within public health, or to carry out related empirical research. The answers to these questions are not at present clear (4, 12). If certain beliefs and ideas are not welcome, it would be good to have greater clarity on which are considered inadmissible. Beyond the question of what is admissible, there is an additional question as to whether alternative viewpoints should in fact be sought out, both for the sake of intellectual diversity and the advancement of knowledge, and also for the sake of representation. The research at many schools of public health is predominantly supported by federal grants, publicly funded by taxpayers. To what extent should the diversity of viewpoints within the general public not be only permitted, but even actively represented, within academic public health?

These concerns are not merely academic or theoretical. Schools of public health train and shape a nation’s future leaders. On controversial issues concerning abortion or same-sex marriage, for example, roughly 30–50% of the United States’ population hold positions contrary to those often presumed in public health and medicine (13, 14). Such groups thus constitute 100 million or more people, in the United States alone. To what extent are we equipping future public health leaders and academics to deal with this diversity of viewpoints? To what extent are we providing an environment in which to even *understand* different viewpoints? Excessive protection from ideas and people with whom one disagrees can make a person psychologically weaker (15) – weaker in understanding and knowledge, less able to find common ground, and less able to serve the entirety of one’s country and world. Moreover, if individual leaders and organizations in public health are seen as overly partisan – and not committed enough to understand the concerns of others – then trust in public health institutions will likely continue to erode. This may ultimately often gravely compromise the capacity of these institutions to promote population health.

We need a robust free exchange of diverse positions so that we can engage civilly and thoughtfully in society, and so that we can find common ground. To facilitate this, schools of public health could, for example, adopt the following practices: (i) implementation of training on the positive value of academic freedom and the free exchange of ideas; (ii) regular data collection on whether students, staff, and faculty feel comfortable sharing what they really think about controversial issues, both inside and outside of the classroom; and (iii) the introduction of seminars on understanding diverse intellectual viewpoints, which would bring together speakers on different sides of an issue to model civil discourse, to help us uncover differing presuppositions and values, and to hopefully find common ground.

Schools of public health could also sometimes consider intentionally hiring faculty who conduct research with viewpoints on important public health issues that constitute minority positions within the field but correspond to the positions of large portions of the population. The autonomy of the faculty in shaping a discipline should be respected, and hiring practices arguably should not be imposed by governments or funding bodies (16–18). However, both for the advancement of knowledge and of population health, a faculty might sometimes freely choose to hire in research areas that are constitutive of important minority positions with respect to an academic institution. We could moreover also better recognize the intellectual diversity that is already present and, by encouraging freedom of expression and a free exchange of ideas, make use of such diversity. Schools of public health have long tried, and often continue to strive, to achieve balance between the pursuit of knowledge, critical reasoning, and the promotion of policies that further justice, equity and public health. Each of the above practices could help foster a healthier academic community, greater respect for intellectual diversity, and greater capacity to work together for the common good.

## Discussion: navigating our disagreements in academic public health

Many faculty at schools of public health always have and always will have a strong commitment to public health advocacy. However, there are complexities with regard to how best to carry this out in the context of a pluralistic society. Different communities – whether LGBTQ+ communities, or different religious communities, or different political communities – will have different values, and different understandings of what is good. Questions concerning means and policies can, to a certain extent, be addressed by empirical research. But questions concerning values, and the nature of well-being, cannot. Within a pluralistic society, we can try to empower different communities to pursue the values and ends that they deem most important. These distinctive values will, however, inevitably sometimes come into conflict, and we might also fundamentally disagree on the appropriate policies and means. Our democracy provides a system to adjudicate between differing positions. However, these adjudications will not always go in our preferred manner, and everyone will likely be somewhat dissatisfied with regard to how certain aspects of that democratic process plays out.

An overemphasis and focus on our disagreements will often lead to greater conflict. It is not that these disagreements do not matter, but there is a question as to how much emphasis they are given. Are they the central focus of our political energies, or are these important but auxiliary topics with respect to our interactions with others? Ultimately, we need a genuine mutual respectful acknowledgement that we do not agree on all things, including very important issues. Through civil discourse and a free exchange of ideas we can, however, understand each other’s values and priorities more fully. We can come to understand that reasonable people of goodwill can disagree on important matters. We can also better see where there might be common values. Such common values arguably extend to a number of aspects of individual and social well-being including happiness, health, meaning, character strengths, relationships, and financial stability, all pursued in a just and equitable manner (19, 20)(VanderWeele et al., accepted manuscript)^1^. Even amidst disagreements, we can meaningfully work together to pursue policies that promote values held in common. A more robust free exchange of ideas, values, and viewpoints, carried out civilly, has the capacity to highlight our agreements and common pursuits, and to respectfully acknowledge and try to navigate our disagreements. Academic institutions should take the advancement of skills to work together, across differences in moral systems, identities, and values, as a critical part of preparing leaders and academics to promote the common good.

Even when a policy or political determination proceeds in the manner we think best, there still needs to be a realism as to what political action can actually accomplish. A policy or change in law can grant new freedoms, and rights, and responsibilities, and can restrain or enable action and behavior in various ways. However, the effect of a change in policy or law on beliefs and values is more complex. Policy and law will influence beliefs and values, but beliefs cannot be enforced by law. Law will also often not alter the beliefs and values of a particular community. Shame is sometimes used to bring about such alterations, and this can sometimes be effective. However, it can also be resented, and sometimes only alters what people are willing to say they believe, rather than what they actually believe. Shame is also less likely to alter values and beliefs that are embedded within a community’s life or that are rationally grounded. For those to change, rational discourse and persuasion, as well as consideration of a community’s lived experience, will often be needed.

There are reasonably well-defined categories of speech that fall outside of constitutional protections (21). Schools of public health, at least at private universities, are in principle free to restrict those yet further. However, as noted above, this might well compromise our capacity to pursue knowledge. There is also arguably a danger in such restrictions with regard to our capacity to work together, a danger that is well-characterized in an address of Frederick Douglass (22): “Liberty is meaningless where the right to utter one’s thoughts and opinions has ceased to exist. That, of all rights, is the dread of tyrants. It is the right which they first of all strike down.”

The only way that we can have true inclusion and belonging for everyone –the LGBTQ+ community, but also for Christians, Jews, Muslims, liberals, conservatives, and all others– is a radical openness to the free exchange of ideas. This can be carried out respectfully and civilly, but ultimately we need to accept that many others will disagree with us. Different people have different moral understandings about right and wrong; we may find some ideas painful and hurtful. Most nontrivial ideas about policy will likely be disadvantageous, hurtful, or offensive to at least some. However, a diverse range of viewpoints and actions are protected within our constitutional order and are within the bounds of academic freedom. Our democracy and universities should be able to sustain such diversity and disagreement. This does not mean that various moral positions should not come under scrutiny. On the contrary, there should be open disclosure and debate of moral systems, values, identities, and their grounds. This will again enable a better understanding of others’ and our own perspectives, and also opportunities both for reasoned persuasion and for finding common ground.

The alternative for academic public health to a more radical openness to a free exchange of ideas is to exclude, or silence, or suppress, alternative ideas, beliefs, and moral principles. One might take the position that Christians, Jews, Muslims, conservatives, and others are welcome so long as they either hold majority public health positions or remain silent on certain issues. That may work, and perhaps to some extent has worked, at schools of public health. However, it is not similarly an option for our society. While the proportion of Americans identifying as liberal has increased over past decades, this figure still stands at only 25%, in contrast with 37% of Americans who describe their political views as moderate, and 36% as conservative (23). With regard to religious identities, world-wide there are approximately 2.4 billion Christians world-wide, 1.9 billion Muslims, and billions of other faiths (24). Their beliefs are diverse, but many hold the positions that seem to be considered unacceptable within academic public health (4). Schools of public health have the option of working to oppose, suppress, and silence those beliefs; or may hope to change or convert them; or may acknowledge the disagreements and nevertheless find ways to work together in our various societies across the globe. The distribution of views of academics within schools of public health on controversial moral issues is very different from the diversity one finds worldwide, and it is not clear that this is likely to change. Some projections, for example, suggest that the proportion globally who identify as religious will increase over the coming decades (25).

There are numerous examples of partnerships between public health institutions and religious organizations on working towards common ends, even when there is deep disagreement over values (26–31), and various other examples of working together on public health issues across political lines (32–40). However, it is not clear that this is the dominant model of interaction at present. While suppression of viewpoints is a real option within academic public health, it is less clear this will be effective in society more broadly. It seems that there we are faced with only the options of either increasingly vitriolic fighting, or of attempting greater civil discourse, attempting to find common ground amongst our pluralistic perspectives, and accepting that the democratic process will sometimes not turn out as we like. The question then arguably arises as to which of these two approaches will schools of public health ultimately contribute. The relative balance of its contributions could make a great deal of difference to the future and well-being of our democracy. It is possible different schools of public health may choose to move in different directions. However, it seems that this is a discussion worth having.

## Data availability statement

The original contributions presented in the study are included in the article/supplementary material, further inquiries can be directed to the corresponding author.

## Author contributions

TV: Conceptualization, Writing – original draft, Writing – review & editing.

## Funding

The author declares that no financial support was received for the research, authorship, and/or publication of this article.

## Conflict of interest

The author declares that the research was conducted in the absence of any commercial or financial relationships that could be construed as a potential conflict of interest.

## Publisher’s note

All claims expressed in this article are solely those of the authors and do not necessarily represent those of their affiliated organizations, or those of the publisher, the editors and the reviewers. Any product that may be evaluated in this article, or claim that may be made by its manufacturer, is not guaranteed or endorsed by the publisher.

## Footnotes

^1^VanderWeele TJ. *A Theology of Health*. Notre Dame, IN: University of Notre Dame Press (2024).
